# Height of Male Prisoners in Santiago de Chile during the Nitrate Era: The Penalty of being Unskilled, Illiterate, Illegitimate and Mapuche

**DOI:** 10.3390/ijerph17176261

**Published:** 2020-08-28

**Authors:** Manuel Llorca-Jaña, Javier Rivas, Damian Clarke, Diego Barría Traverso

**Affiliations:** 1 Escuela de Administración Pública, Facultad de Ciencias Económicas y Administrativas, Universidad de Valparaiso, Valparaiso 2340000, Chile; diego.barria@uv.cl; 2 Proyecto Anillos, Facultad de Ciencias Económicas y Administrativas, Universidad de Valparaiso, Valparaiso 2340000, Chile; javier.rivas@uv.cl; 3 Departamento de Economía, Facultad de Economía y Negocios, Universidad de Chile, Santiago 8380000, Chile; dclarke@fen.uchile.cl

**Keywords:** anthropometry, Chile, prison records, human capital, inequality

## Abstract

This article contributes to the study of inequality in the biological welfare of Chile’s adult population during the nitrate era, ca. 1880s–1930s, and in particular focuses on the impact of socioeconomic variables on height, making use of a sample of over 20,000 male inmates of the capital’s main jail. It shows that inmates with a university degree were taller than the rest; that those born legitimate were taller in adulthood; that those (Chilean born) whose surnames were Northern European were also taller than the rest, and in particular than those with Mapuche background; and that those able to read and write were also taller than illiterate inmates. Conditional regression analysis, examining both correlates at the mean and correlates across the height distribution, supports these findings. We show that there was more height inequality in the population according to socioeconomic status and human capital than previously thought, while also confirming the importance of socioeconomic influences during childhood on physical growth.

## 1. Introduction

The anthropometric history of Chile has now been well covered thanks to a plethora of articles recently published since the pioneering study of Nuñez and Pérez [1] five years ago, and covering an extensive period of cohorts born from the 1740s to the 1990s [2,3,4,5,6,7,8]. Most of these studies rely on data from army (and navy) records and to a lesser extent from health surveys [2,8] and school records [1]. The studies based on army records, which are of adult males only, are particularly robust and help to assess the long-term evolution of height in Chile in as much as their height data stemmed from general conscription, so that there was neither selectivity in the sample nor significant minimum height requirement, thus avoiding most of the recent criticisms on sample-selection biases rightly highlighted by Bodenhorn et al. [9].

The extant anthropometric history studies on Chilean adult males do not contain much information on the role of socioeconomic status (SES) as a determinant of height, which is unfortunate because “higher SES allows for better nutrition, better health care, reduced physical labor for children, and greater growth-promoting psychological stimulation from parents, schools and peers” [10]. This gap to our knowledge is notorious, in particular if compared to the available studies on Mexico [11], Colombia [12], Brazil [13] or Argentina [14] (three examples within Latin America), including the impact of human capital on adult male height.

This gap in our knowledge of Chilean height is surprising given the fact that the role of SES variables on height has attracted increasing attention within the literature (e.g., [15,16,17,18]). It is now widely acknowledged that there is a strong connection between socioeconomic conditions and human growth at a household level, and in particular that differences in average height by SESs are useful indicators of inequality in the biological standard of living [17,19,20]. This is important for Chile given the recent unrest of October 2019, due primarily to inequality. With regard to disposable income distribution, the country enjoys the sad record of being the most unequal OECD country [21].

The variables used in the regression analysis of previous studies on adult height in Chile include skin color, eye color, region of birth and the urban/rural background of the recruits. Occupation prior to joining the army was also used in some of these studies (as an indicator of socioeconomic status), but this variable was barely documented since most recruits were too young to have a previous occupation other than being students or “just” soldiers, or it was not entered by recruitment officials in the conscription records (another way of looking at it is that conscripts were too young to know how they would earn their living after serving in the army [18]). The impact of previous occupation on adult height is only mentioned in passing for Chile. The only proper human capital variable used so far was a crude measure of literacy: the ability to sign, and, again, it was not always documented. Previous studies may be more representative of the overall Chilean population than our sample is, but they lack information on some important determinants of adult height, in particular social status information. Although useful, most of the variables used in previous regression analyses accounted for a homogenous Chilean adult male population, showing a low level of height inequality, which was surprising, given the extant evidence for other Latin American countries. The previously selected variables explained only a small part of the height variation in the samples.

The main contribution of this study is to provide better or newer information on the impact of socioeconomic status (including human capital) on adult male height, making use, for the first time for Chile, of prison records. Previous studies were mainly concerned with the long-term evolution of adult male height. Although we are not analyzing the trend of mean heights through birth decades, our data also show height stagnation during the 1880s–1910s and a slight decline during the 1920s–1930s. This is very much in line with the main findings of the previous studies [5,6,7].

The article is divided into four more sections. The literature review is followed by an explanation of methodology and the nature of our sources and of our sample. This is followed by an account of the Chilean prison system, and in particular of Santiago Public Jail, to better understand the sort of people who ended up falling within our sample. We then present a regression analysis to analyze the impact of some determinants of adult male height in our sample, before concluding.

## 2. Literature Review

There is wide agreement that height is highly influenced by socioeconomic variables such as education, occupation and disposable income [8,20,22,23,24,25], and that in turn height is sensitive to the distribution of income and other inequality measures. Difference in height according to social class is a useful indicator of inequality in biological welfare [17,20]. Systematic height differences have been found by variables such as occupations and skin color across populations, in particular before the mid-twentieth century [16,20,24,25]. Huang et al. [23], for example, found large differences in individuals’ statures according to their education level and their parents’ occupations for Dutch conscripts born during the 1940s. Schoch et al. [26], analyzing Swiss recruits born during the 1850s–1940s, found that social-class affiliation was the most important determinant of differences in height. Finally, in terms of European examples, Ayuda and Puche-Gil [27] found that literate conscripts born in Spain during the 1870s–1950s were almost 1 cm taller than illiterate ones.

Thus, our new data on SES for Chile are very useful since we can reasonably assume that adults with higher SES (e.g., measured through more skilled occupations and/or higher literary levels, having been legitimately born and having white skin color) were more likely raised within households with higher income, therefore enjoying better nutrition, ceteris paribus. Height and income are usually closely correlated [28], but not always. In turn, height inequality tends to correlate with income inequality [29] (for a recent review of these topics, see [15,30]). This is important because it was highlighted by Steckel that stature in developing countries is very useful to monitor the population’s health status [17,20].

Indeed, family income is regarded as a reliable predictor of adult stature [18,20,31], despite some well-known puzzles. There is also evidence that better educated parents usually lead to healthier and taller children due to the positive impact of better-informed childcare practices. Educated parents are able to provide better standards of hygiene and to provide a more nutritious diet, even controlling for income. Finally, it is also believed that the presence of the father, apart from the mother, has a positive impact on children’s height (for a summary of research on these topics, see [17,18]).

However, before this work, we knew little about the relationship of these variables for Chile’s adult males. We were under the erroneous impression that height inequality was less substantial than in other Latin American countries. The only exception would be the work of Viviani et al. [8], who correlated the height of urban male workers with their education level (i.e., “primary”, “secondary”, “technical” or “university” education) for a small sample (1400 workers) of cohorts born during the 1960s–1990s, finding that workers with primary and secondary education were shorter than the rest. For example, those with university degrees were between 3 and 5 cm taller than those with primary education only, depending on the decade of birth. They also found that educational level (as defined in their sample) may no longer be associated with height or socioeconomic situation in adults with technical or university education. Thus, Viviani et al. [8] called for the inclusion of other SES variables to establish the socioeconomic background of subjects, not only this rough measure of educational level, which we do here. When analyzing Chilean boys rather than adult males, Núñez and Pérez [1] found SES differences in heights of 9–11 cm up to the late 1940s. However, after the 1930s, there was substantial convergence in height among socioeconomic groups, so that SES differences in height decreased to 5 cm by the 1990s.

For other Latin American countries, for example Mexico, population height with and without passport was used as a proxy for upper and lower SES, respectively [11], finding that those born during the 1900s, who had passports, were some 3 cm taller than military recruits. For Argentina, Salvatore [14], used years of schooling as a proxy for human capital, finding that prisoners with 7–10 years of schooling were 1.7 cm taller than those with 0–3 years of schooling. For Colombia, Meisel et al. [12] found that, compared with individuals with primary education, those with secondary education were 1% taller, technicians 2.4% taller and university students 2.5% taller. Similar differences were found for Brazil [13]. These studies also used occupation (e.g., professionals, white-collar workers and skilled and unskilled workers) as a proxy of SES, finding that there were differences of between 1 and 3 cm (according to decade of birth) between unskilled workers and professionals for Mexico, employees were 1.2–1.6 cm taller than unskilled laborers in the Argentine case, while and differentials between skilled and unskilled Colombian workers were 3 cm on average for men born during the 1920s–1980s. Other recent studies are those of López-Alonso and Vélez-Grajales [32] and Martínez-Carrión and Salvatore [33], which provide an overview for Latin America. López-Alonso and Vélez-Grajales in particular found that Mexicans with no education were nearly 7 cm shorter when compared with those with university degrees.

Prison records have been used for biological and medical research for over a century in countries such as Britain and the United States [34]. They have also been widely used in anthropometric studies in particular for many countries, including Britain, Ireland, the US and New Zealand (e.g., [35,36,37,38]). Within the region, prison records were used in other Latin American countries, e.g., by Carson [39] for Mexico, Frank [40] for Brazil, Baten et al. [41] for Peru (and Brazil too) and Salvatore [14] for Argentina. However, despite these studies, penitentiary records represent a comparatively underused source of anthropometric history [14,42].

Nonetheless, it is worth mentioning the main results found by previous studies based on prison records regarding the impact of SES on height. For the US, Tatarek [42] found small height differences for Ohio convicts born during the 1780s–1840s, although the author could not include variables such as education and occupation, which she thought had greater influence on height than place of birth. Maloney and Carson [35], analyzing cohorts born in Ohio during the 1810s–1890s, found that black men were shorter than white men (although not by much), reflecting the poorer and less healthy living conditions faced by African-Americans throughout the 1800s. Similar results were found for Tennessee convicts in the 1830s [43]. For Canada, Arsenault-Morin et al. [44] used records of the Quebec prison for cohorts born during the 1780s–1820s, but the authors could not find information on socioeconomic variables, focusing on international comparisons instead. Nicholas and Oxley [36], who focused on women in England and Wales, found that the height of rural-born women fell more than that of urban-born women during the early nineteenth century. In mid-nineteenth century Ireland, prisoners declaring an ability to write were considerably taller than those who did not [37].

For New Zealand, Inwood et al. [34] found that socioeconomic variations in height were pronounced for those born in the 1880s and 1890s, but that a century later these differentials had been reduced. Overall, they found that inmates who were professionals were some 1.5 cm taller than those who were laborers, and the Pākehā people were about 1 cm taller than the Māori. Within Latin America, Baten et al. [41] used convict records for Brazil, including occupation and skin color, for birth cohorts of the 1810s–1880s. They found modest differences in height between unskilled and skilled workers, with the exception of professionals, who were some 3 cm taller than unskilled workers. Frank [40] showed that there were significant changes in the heights of prisoners in Brazil according to race, and skilled workers were slightly taller than unskilled ones around the mid-nineteenth century. The same study by Baten et al. [41] used a prison sample for Peru, for cohorts born during the 1820s–1880s, finding that there were no significant differences in height by occupation, but that there were large variations in height by ethnicity: indigenous people were shorter than whites by some 5–6 cm. In turn, Carson analyzed height differences for Mexican inmates according to occupation, concluding that, of those born during the second half of the nineteenth century, Mexican farmers were taller than white-collar and skilled workers.

## 3. Methodology, Sources and Nature of Our Sample

We are conscious of the fact that prison inmates rarely represent the overall population of a country: there is usually a height bias and an occupation bias (on selectivity issues of prison samples, see the works of Baten et al. [41], Carson [45], Salvatore [46], Nicholas and Oxley [36], Maloney and Carson [35], Inwood et al. [34] and Ó Gráda’s [37]; among many others). Most of the time, prison samples are more representative of the lower ranks of society [47], rather than of the whole population: the poor have less means to protect themselves in judicial courts and are also more prone to enter into criminal activities [9,35,44,45]. It is believed that the average height of prison samples is smaller than that of the overall population, although not by much since greater than average height facilitates violent criminal activities [31]. Furthermore, there is also a geographical bias in those cases where the prison data belong to a narrow geographical area, as in our particular case, since there is an over-representation of people from an urban setting: Santiago, the capital of the country (52% of our sample was born in Santiago, 7% in the north, 5% in the south and the rest in the center of the country excluding Santiago). In contrast, in the 1907 census, only 16% of the population resided in Santiago (Baten et al. [41] faced the same issue for Peru). Despite these shortcomings, it is also acknowledged that prison samples contain precious information unavailable in other sources, while they do not suffer from issues related to the minimum height requirements of the army, which is an advantage [35,39,42]. Hence, it is always advisable to contrast results based on military population against those of civilian records [48]. For the period under study (i.e., those born between 1882 and 1934), the raw average height for those aged 20–55 was roughly the same for both sources: 167 cm.

As far as human capital is concerned, our database contains a better measure of literacy than previous studies, since inmates were classified into three categories: illiterate, able to read only (a few cases only), and able to both read and write. Regarding other socioeconomic status measures, apart from previously used variables such as skin color (as an indicator of ethnicity), we have a variable never used before for a Latin American sample: the classification of a son at birth, i.e., legitimately born and illegitimately born (which was subdivided into “illegitimate” and “natural son”, or “un-recognized”). “Legitimate son” was someone born to married parents (or whose actual parents married later on, and recognized him as their own son). All other children were classified by Chilean law as “illegitimate sons”. In turn, “illegitimate sons”, according to the Civil Code of 1855 (in force until 1998), could then be subdivided into “just illegitimate” or “natural” (also illegitimate). In the latter case, the son was born to an unmarried couple, but either the mother or the father recognized him (usually his mother). Those classified as “just illegitimate” were unrecognized children also born to unmarried couples, more often orphans. In our regressions, we merged both categories of illegitimacy into a single one.

Although new for Latin American studies, Baten and Murray [49], in a pioneer article, explored this topic for Bavarian women (see also [50]). We can reasonably assume that legitimately born children were raised within households with higher per capita income. Those recorded as illegitimate sons probably grew up in households headed by single mothers from lower socioeconomic groups. It was far less common to be a single mother in Chilean upper classes during the nitrate era since it was perceived as a shameful situation [51]. It has been found, for example, that during our period of study child mortality among illegitimate children was far higher than amongst legitimately born children [52,53].

We also classified prisoners’ surnames by ethnic origin (e.g., British, German and Italian), to contrast with Spanish (Castilian) surnames (most of the population) and Mapuche surnames. This allowed us to study the impact of surnames or ethnic identities on SES, and indirectly on height. A recent study (covering cohorts born during the 1830s–1930s) has shown that Chileans with Northern European surnames had a relatively high rate of numeracy compared to people with Spanish surnames [21]. Many of the Northern European families who emigrated to Chile after independence from Spain (i.e., from the 1820s) worked in trade related activities. Given that Chile’s economic growth during our period of analysis was fueled by booming exports, and that only the economic elite was in a position to take advantage of this nitrate boom [5], to which those of wealthy British and Germans descent belonged, we would expect that those with Northern European surnames, ceteris paribus, enjoyed better biological welfare than the bulk of the population. In other words, the substantial profits generated by booming exports did not filter down to benefit the population evenly.

Likewise, the Mapuche, who were conquered by the Chilean state during the 1860s–1870s, were incorporated into Chilean society under difficult and discriminatory working and living conditions. For example, in 1941, Israel Drapkin and Eduardo Brücher maintained that criminality was linked to biological factors: black people were the most violent of all “races”, while Mapuche people were more likely to commit theft. Six years later, a study by Alexis Da Silva, published by *Revista de Criminología y Policía Científica* (an official publication of the Chilean National Prison Directorate), maintained that crime in Chile was deeply rooted in the Mapuche inheritance: the Mapuche were perceived as intrinsically criminal, with poor morals and inclined to alcohol consumption, which in turn fueled crime [54].

Thus, we would expect prisoners with Mapuche surnames to be shorter than the rest, in particular for our period of study, since it was the most difficult time for Mapuche people: their land had been recently expropriated and they were relocated in poor quality lands or forced to emigrate. Chilean upper classes have been historically composed of white people, most of them with British, German and Basque surnames, as a result of the permanent inequality of the country and its limited social mobility. According to data recently published, which considered 8 million Chilean people, amongst the 50 most common surnames of the most prestigious professions in Chile, an overwhelmingly majority were Basque, British, German, French and Italian, while the 50 that did not even enter that list (with more than 200 observations) were mainly Mapuche [55] (on this, see also the works of Núñez and Pérez [56] for Chile and Clarke [57]).

More importantly, over 99% of our sample reported a previous occupation before entering jail. This is the first study for Chile where that variable has been so nicely documented, showing a massive height difference between professionals (those with a university degree, such as lawyers, engineers, teachers and accountants) and the rest. This is perhaps the first time we have sound data to distinguish members of the Chilean economic elite from the rest of the population, since during our period of study only a few had the means to complete university studies.

All of our data come from Santiago Public Jail records (*Cárcel Pública de Santiago*), currently available at the National Archives, Public Administration’s collection (Archivo Nacional de Chile, Archivo Nacional de la Administración, Colección Cárcel Pública, Inmates Records, volumes 190, 276, 277, 302 and 303). This was Chile’s main prison for most of the twentieth century and certainly during the totality of our period of study. We managed to find five large volumes of the prison’s inmate records (*Registro de Detenidos*), which contained data for 21,506 prisoners of this famous jail that,, after eliminating duplicated names (repeat offenders, for whom we entered their first registered height), foreign born inmates (so that we can attribute height entirely to nutritional and environmental conditions in Chile) and those under 17 years (still growing) or over 55 years (shrinking), was reduced to 20,325 observations, still a respectable sample size.

We did not sample but rather collected all surviving data within these five volumes. To the best of our knowledge, there are no more surviving volumes of the Public Jail (or any other) at the National Archives that contain height, which is unfortunate, as we are not able to state how many volumes have gone missing. We can only state that our sample is probably a subsample of all prisoners at Santiago Public Jail during 1936–1952. According to the Chilean Statistical Yearbooks (*Anuarios Estadísticos*), between 1936 and 1952 there were around 556,000 entries to Santiago Public Jail (on average around 34,000 per annum), most of them for a short stay. We do not know how many of these were repeat offenders, so as to estimate what proportion our 20,325 sample is of the total number of inmates that circulated at this jail during our period of study, but it is certainly higher than this minimum 4%. However, we compared our data to those for all prisons in Santiago (available at aggregate level from the Chilean Statistical Yearbooks), and, for our period of study, 60% of all inmates in Santiago were in jail for theft. In our sample, this share is very similar: 57%.

The process of transferring files from the Chilean prisons service to the Chilean National Archives is undocumented for that period of time, or any other. We endeavored to get more records directly from the Chilean national prison service (*Gendarmería de Chile*), but without success. We were denied access to such records twice, on account of the need to maintain the confidentiality of the inmates (on data protection grounds, even for those deceased).

However, our sample size is the largest for any published work based on prison records for any Latin American country. The recent study published by Salvatore [14] for Buenos Aires used fewer than 9000 observations, Frank [40] for Brazil used just over 1000 and Baten et al. [41] used fewer than 7000 for Brazil and just over 1000 for Peru. Beyond the region, our sample size is comparable to previous studies on developed countries: Carson [45] in the US used 20,000 observations [35] and also used for the US nearly 30,000 observations but for a longer period of time; Ó Gráda’s [37] sample contains 3000 observations only for males for Ireland; Baten and Murray [50] used 4000 for Bavaria; Arsenault-Morin et al. [44] considered nearly 12,000 for Canada; and Inwood et al. [34] used around 26,600 for New Zealand, also for a longer span of time. Overall, comparatively, we have a good sample size. It is perhaps the largest on average per birth decade within prison sample studies.

These five volumes contain entries to Santiago Public Jail between 1936 and 1952. Therefore, all the data belong to prisoners born during the 1880s–1930s (from 1882 to 1934 to be more precise), a period known as the nitrate era in Chilean economic history, due to the massive exports of nitrate that generated the first export-led growth episode of the country’s history. Likewise, the period ca. 1936–1952 (when offenders committed the crimes for which they were incarcerated), is a short period of time, belonging to the first period of industrialization led by the state, which was also stable (the negative impact of the 1929 depression had already passed). We are lucky that all surviving records belong to men born during a specific period of Chile’s economic history, and that they committed their crimes also within a short period of time (sixteen years only), again a very specific period and without major economic turbulence.

This is important because it is believed that the rate of some crimes (for which people ended up in Santiago Public Jail), especially theft, are countercyclical: they increase during difficult economic times, potentially generating a source of selection bias given the fact that shorter individuals (usually poorer than the average population) might be more likely to commit a crime under harsh economic conditions [44,50]. For our period that would not be a major problem since Chilean economic performance was stable during 1936–1952. There was continuous growth: only in 1947 was there a negative growth of real GDP [58].

Regarding the birth period of the inmates, the nitrate era was characterized by a very high level of per capita exports. Chile’s export economy has always been heavily based on minerals, to an extent unequaled in Latin America. The Chilean macro-economy flourished during the nitrate boom. Between 1880 and 1929, Chilean per capita GDP more than doubled. Inflation was, on annual average per decade, below 7% [58]. There were also considerable investments in infrastructure and education, in part funded by increasing fiscal revenues, mainly coming from export duties on nitrate. The public sector increased in size, and with it government expenditure. Finally, the industrial sector slightly improved its share within the whole economy, but there was no structural change in the Chilean economy. There was economic growth, but little development [7,59].

During this period, characterized by a highly organized public sector (in particular if compared to the first decades after independence), at the moment of entering a public jail, the following variables were recorded by prison officers: name and surname, crime committed, height, eyes color, skin color, age, year of birth, marital status, classification of child at birth, number of sons, locality of birth, literacy and previous occupation (this is similar information to that used by other studies working with prison records [48], except for the fact that in other studies the length of the prison sentence is also included (e.g., [42,43]), but was not available in our records). Based on these variables, we added new ones: surname ethnicity, year and decade of birth, province and macro-region of birth (a standard procedure in the literature, e.g. [42]). In this case, we used a more disaggregated geographical classification than the previous studies on Chile, in particular to distinguish those born in Santiago and nearby: *Norte Grande* (Big North, the provinces of the Atacama Desert, Tarapacá and Antofagasta), *Norte Chico* (Small North, comprising Atacama and Coquimbo provinces), Santiago-Valparaiso-Aconcagua (a mostly urban area), Center (from O’Higgins to Concepcion, the most important agricultural zone) and South (all other provinces to the south of Concepcion, characterized by low population density and very rainy weather). We also grouped the hundreds of occupations reported into five large groups: professionals, *agricultor* (farmer), white-collar workers, unskilled manual worker and skilled manual worker. This is a usual practice in the literature using prison records. Although there is no agreement on the number of broad groups of occupations to be used, usually authors prefer to define 4–7 categories. For example, Maloney and Carson [35] merged the occupation data into four categories, while Salvatore [14] used seven categories.

We are confident that the data entered by prison officers was good quality. Prison officers were trained to register anthropometric data of the inmates, as part of a modernization process of the police and the penitentiary sector [60]. For example, from 1900, an anthropometric manual [61] was distributed in all Chilean prisons. It taught the Bertillon system (named after Alphonse Bertillon, 1853–1914, a pioneer in the identification system based on physical measurements), to record physical characteristics of those incarcerated: the aim was to measure and physically describe inmates, in case of runaways. The manual stated that this procedure was similar to that used by the Army, to facilitate and speed up the accurate entry process of anthropometric data (including height), descriptive characteristics and any special mark such as physical “anomalies” or tattoos.

**Figure 1:**
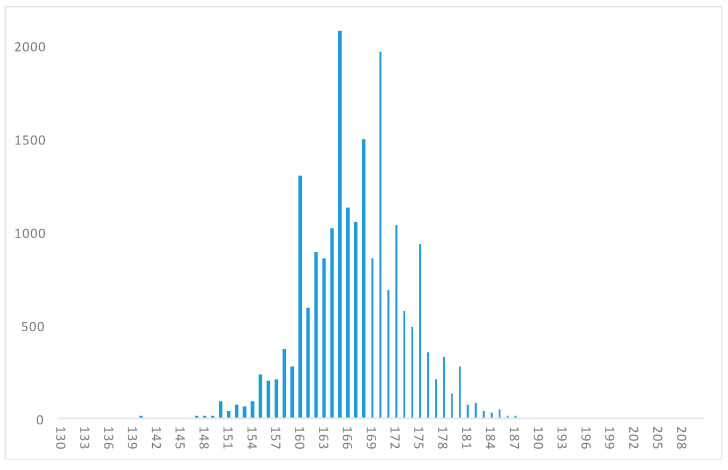
Histogram, distribution of adult male prisoners’ height in Santiago de Chile (aged 17–55), born during the 1880s–1930s (in centimeters, 20,325 observations).

The height histogram of our refined sample (Figure 1) shows an approximately normal distribution, although with the usual heaping at 150, 155, 160, 165, 170, 175 and 180 cm. In turn, given that the inmates in our sample entered jail between 1936 and 1952, and within the province of Santiago, we compared that population to Santiago’s total male population in the national census of 1940, our sample accounting for 4.3% of the total male population of Santiago that year, and around 8% of the population aged 17–55 years.

**Figure 2:**
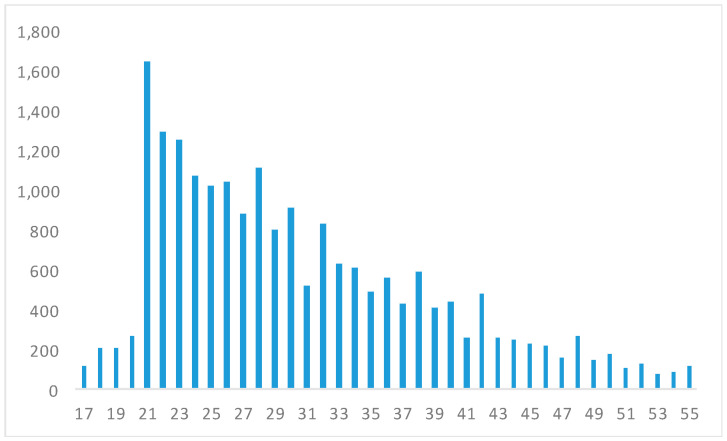
Histogram, distribution of adult male prisoners’ age in Santiago de Chile, born during the 1880s–1930s (in years, 20,325 observations, for ages 17–55).

**Figure A1:**
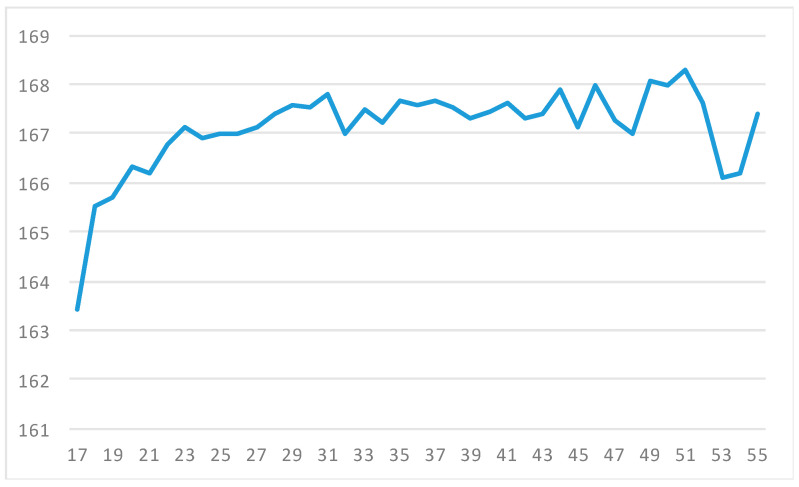
Height (cm) by age (years) profile, Santiago Public Jail.

The age histogram does not show any significant age heaping (Figure 2), while it shows that 80% of our sample was aged between 20 and 39, with only 3% being younger than 20, another 3% older than 49, and the remaining 13% being in their forties. This pattern would not surprise us: the average young age of offenders is typical of most prison records [34,44]. For example, for the whole Chilean male population, according to data from the censuses of 1930 and 1940, around 31% of the nation’s male population was aged between 20 and 39 in both censuses, and around 30% in the 1952 census. In our regression analysis, we controlled by age, in particular for those between 17 and 20 years of age, since they were probably still growing (see Appendix A, Figure A1).

Regarding the descriptive statistics of some of the main variables we entered, it is worth mentioning that those with a university degree were, on raw average, 169.7 cm tall (listed as professionals in Table 1), around 3 cm taller than unskilled workers, this being one of the greatest differences ever reported for social groups in Chile. This is unsurprising as it has been documented that income inequality was increasing during our period of study, despite sustained economic growth [5]. It is intriguing, however, that unskilled manual workers were slightly taller than skilled manual workers, although only by 0.2 cm in raw averages. López-Alonso [11] reported fairly similar differences for Mexico for the 1840s, 1860s and 1880s.

At this point it is worth comparing our data in Table 1 with national census data. For example, in the 1930 census, 5.9% of the population could be classified as professional, far more than the 1.6% of our sample. In that year’s census, 55% of the workforce classified themselves as farmers, far more than the 5.2% of our sample. These two facts confirm the idea that prison samples are more representative of people of lower socioeconomic status, and that in this particular case we have a selectivity bias in favor of people living in urban areas.

As far as literacy is concerned, most of the inmates in our sample were able to read and write (83%), and this group was around 0.5 cm taller than the rest (Table 2). To check how representative our sample is in relation to the whole country’s literacy, we can compare it with the data from the censuses of the first half of the twentieth century. A comparison shows that in the 1930 and 1952 censuses, for example, 75% and 83% of the Chilean population older than 15 years, respectively, was reported as literate. If those older than 16 (as in our sample) are considered, that share would be slightly higher (but not by much). In this respect, our data are fairly representative of the whole country, as far as literacy is concerned.

Eighty-eight percent of the sample was declared as legitimately born, and were around 0.6 and 1.1 cm taller than those reported as natural sons and illegitimate sons, respectively. This is not surprising: natural and illegitimate (natural is a subsection of illegitimate) grew up in households headed by single mothers or in orphanages. A 1933 government report accounted for 70,000 Chilean orphans aged under 18 living in the streets (homeless) [62]. It was also estimated that around this time 80% of the bakeries in Santiago employed orphans, who lived there [63]. Illegitimate sons in particular are known in the Chilean historiography as *huachos*. If compared to census data, there is an overrepresentation of legitimately born children in our sample. The national shares of legitimately born children in the 1880s, 1890s, 1900s, 1910s, 1920s and 1930s were 75%, 68%, 65%, 62%, 64% and 71%, respectively (we collected annual data from Chile’s Statistical Yearbooks (Demography’s section), from 1919 to 1940; the 1919 issue in particular contained data as far back as 1880). Given that this condition was self-reported by inmates, and that some were probably ashamed of their illegitimate condition, they probably tried to hide it. Illegitimate people were highly stigmatized and discriminated against in Chile during our period of study [51,64].

Given that family context is important in understanding stature, since children are dependent on the resources provided for them by their families [10,31,34,65,66], it is worth paying attention to the Chilean *huachos*’ living conditions. It may seem surprising that the raw average differences in mean height between legitimate and illegitimate sons is not that substantial (0.6 cm is still a considerable difference). However, illegitimate children usually had higher infant mortality rates, in large part due to differences in breastfeeding [49], so that those illegitimate children who survived were the stronger ones. This was the case in Chile: there is evidence of very high infant mortality rates among illegitimate children [51]. The 0.6-cm difference in raw average height during adulthood that we report may be consistent with the idea of “scarring versus survival”, if only relatively stronger illegitimate children of the population survived and reached adulthood [49].

Adult height has been regarded as a sound variable for testing whether quality of parental care during early childhood differed between the illegitimate and the legitimate, since illegitimate children were typically raised under straitened circumstances [49,50]. Gabriel Salazar [51] provided a good account of the harsh economic circumstances in which single mothers raised their *huacho* children during the nineteenth century and early twentieth century in Chile, providing insufficient care for their children (including lack of breastfeeding and a poor diet) and most of the time with an absent father [64]. The average personal disposable income of *huachos*’ households was certainly lower than most others.

Additionally, we added a new dimension to our data by classifying the inmates’ surnames by ethnicity. Table 3 shows the raw average heights for those categories with over 40 observations only, excluding Croatian and Arab surnames, for which we had a handful of cases only. As can be seen, most of the sample had Castilian surnames (95%). Of the remaining 5%, those with Northern European surnames (British and German) were the tallest of all, measuring over 170 cm, while those with Mapuche surnames were the smallest group, measuring less than 165 cm. These 5- and 7-cm raw average differences are the largest ever reported for any social group in Chile. Finally, as far as skin color is concerned, *trigueños* or sun-burned mestizos (a few cases only) and whites were the tallest of all, measuring around 167.5 cm, some 0.8 cm taller than those with dark skin color, in line with evidence from previous studies based on army records. Unfortunately, for this variable, we could not gather information for 47% of the sample (Salvatore [14] had a similar problem for Buenos Aires).

We grouped all crimes into seven broad categories. The most common crime for which people were arrested was theft (57% of all cases), which once again suggests that our sample is mainly composed of members of the lower classes who were compelled to steal (surely for economic reasons). It was followed by those causing physical injuries (17%), white-collar crimes (usually linked to financial affairs) and homicides (Table 4). As was to be expected, inmates imprisoned for committing white-collar crimes were the tallest of all (indeed, half of all professionals in our sample were jailed for this sort of crime), while those committing robberies were the shortest of all.

## 4. The Chilean Prison System and Santiago Public Jail

Before going to our regression analysis, we wanted to illustrate the nature of Santiago Public Jail, as well as the Chilean prison system. A few decades after independence, Chile, as with most other Latin American new republics, started to implement a set of measures to “civilize” the prison system [67]. The overall condition of the prison service was hotly discussed in parliament and the national press. As a response, one of the measures taken was to centralize the prison system under a unique national authority. The year 1889 marked the creation of the National Prison Directorate (*Dirección General de Prisiones*), which was in charge of running the sector until 1896, when the Justice Ministry took over. Between 1930 and 1932, the National Prison Directorate was refounded, including the establishment of a Vigilance Prison Service [68,69].

The capital city had three prisons during the nineteenth century: the Penitentiary (*Penitenciaría*), the Preside (*Presidio*) and the Public Jail (*Cárcel Pública*). However, this infrastructure was insufficient for an increasing population, while overall conditions were poor, so that, under Balmaceda’s presidency (1886–1891), 18 new prisons began to be constructed nationwide. One of them was the new Santiago Public Jail, which was begun in 1887, with operations beginning in 1891 [68].

Apart from working on increasing the capacity and improving the living conditions of the inmates, the government also designed a prison policy to differentiate the role to be accomplished by each sort of prison. For example, all prisons under the umbrella of the National Prison Directorate were for males only. Female inmates were under the custody of the *Buen Pastor* nuns from 1860 and for nearly a century. The state guarded male inmates only, while convicted females were left to the Catholic Church [69,70].

From the late nineteenth century, the role to be played by each of the men’s prisons was clearly established within the national prison system. The Penal Code of 1874 made clear distinctions among penitentiaries, presidios and jails. Jails in particular hosted those definitely condemned to prison (articles 86 and 87). The Prison Regulation of 1911 continued in the same vein, classifying the prison establishments in penitentiaries, presidios and jails, but this time jails also contained those subject to preventive imprisonment from 1911, according to article 312 of the Code of Criminal Procedure of 1906. Additionally, in 1928, a new Prison Regulation established that any convict with less than four months of his sentence remaining could be transferred to a jail or a presidio, including the Santiago Public Jail [69,71].

Santiago Public Jail, in the period covered by our sample, received a wide variety of inmates, and at increasing rates. In 1906, it hosted around 700 inmates (on 31 December of that year); by the mid-1940s, this number had increased to around 1100 prisoners [68,70], reaching a peak of over 1800 in 1949 (Statistical Yearbook of 1949). A point worth stressing is that, throughout the whole period 1936–1952, in which all data of our sample were recorded, there was no institutional change in Santiago Public Jail: it accepted all offenders, for all crimes committed during the whole period. It also remained the most important penitentiary of the whole country, not only of the capital city, hosting around 18% of all inmates of the national male prison system between 1936 and 1951 (Statistical Yearbooks 1936–1951 and [58]).

## 5. Regression Analysis

We conducted formal regression analysis of the measured heights, examining conditional correlations related to the stylized facts documented up to this point. In this analysis, we considered: (i) average conditional correlations in the observed population based on OLS regression; and (ii) how these correlations vary over the full distribution of height using quantile regression. Given the broad academic literature suggesting that returns to anthropometric indicators are nonlinear (for example, see the discussion provided by Baker and Cornelson [72] with respect to height), this suggests that nonlinearities in correlates of height are an important factor beyond mean impacts in the population. We first estimated the following linear regression model (Equation (1)):

*Height_ir_ = α + Demographics′_ir_β + Occupation′_ir_γ + Crime′_ir_δ + μ_r_ + ε_ir_* (1)

where the height of individual *i* from region *r* is regressed on observed characteristics. These characteristics consist of a vector of demographic controls, a vector of occupational group indicators, a vector of indicators for the type of crime committed leading to individual *i*’s imprisonment and regional fixed effects. A stochastic (unobserved) error term is indicated as *ε_ir_*. These controls were entered progressively into the regression model, allowing us to examine coefficients conditional and unconditional on the full set of controls. We discuss the full set of variables in each vector of controls when interpreting results below. Model 1 was initially estimated by ordinary least squares, and then using quantile regression, where we examined the conditional correlation between each variable and height at various quintiles of the distribution of height. In this case, coefficients are presented graphically, along with 95% confidence intervals estimated using a standard bootstrap resample.

Regression results corresponding to equation 1 estimated by OLS are presented in Table 5. In Column 1, only demographic controls are included, consisting of a control for the individual being aged under 21 years (i.e., 17–20), the individual’s legitimacy status and indicator for whether they know how to read or write, their race and their ethnic background as inferred from the individual’s last name. Here, we observe, unsurprisingly, that those aged under 21 years (and hence still growing) are around 1.7 cm shorter than average, holding constant other demographic factors. We similarly observe an “illegitimacy penalty”, where individuals reported as being illegitimate are around 0.5 cm shorter, all else held constant. Individuals who are illiterate are estimated to be a somewhat smaller, 0.2 cm shorter in Column 1, capturing in part socioeconomic differences in access to education. We observe evidence of a gradient by skin color, with black individuals being approximately 1.5 cm shorter than the omitted base category of no reported skin color, and those with mestizo skin being 0.6 cm shorter on average. Finally, the largest conditional correlation is observed among ethnic groups. Those with indigenous (Mapuche)-sounding last names are a full 2 cm shorter than those with Spanish-sounding last names, while those with other European-sounding last names are a full 2 cm taller than those with Spanish last names. In Column 2, we include occupational class dummies, again observing variation, likely in part capturing socioeconomic differences in access to different labor markets. Compared with the baseline category of “unskilled manual laborer”, agricultural workers, professional workers and white-collar workers are all 0.7–2.5 cm taller, while, somewhat surprisingly, skilled manual labors are slightly shorter (0.3 cm). Column 3 additionally adds fixed effects for the type of crime committed by the individual. The only consistently observed fact here is that those who commit white-collar crimes are estimated to be 0.8 cm taller than those committing other crimes, once again in part proxying for socioeconomic factors which are also linked with height. Columns 4 and 5 examine the robustness of these results to controlling for geographic factors (regional fixed effects) and more flexibly capturing age effects (Column 5). Across specifications, all estimates are quite stable, with only some marginal changes in significance of a small number of coefficients, suggesting the correlates documented here are not simply proxying geographic or age characteristics of the penal population of Chile. We similarly observe in Table A1 that these results also hold when conditioning on decade of birth fixed effects. 

Table 5 suggests a clear shift in intercepts between ethnicity groups; however, it does not allow us to separately examine whether the correlates of height in this population may be substantially different by the ethnicity status. We consider this in Table 6, where we estimate the same linear regressions but now separately on each subsample of Mapuche (Columns 1–3), Spanish (Columns 4–6) and Other European (Columns 7–9) individuals. It is important to note that, despite the large total population, the population of Mapuche individuals is small, at only *n* = 87, and as such regression models should be interpreted with this in mind for this group. Nevertheless, across columns of Table 6, it is clear that the correlates of adult height are considerably different depending on the ethnic origin of the individual. For example, consider the case of illiteracy. In both Spanish and European individuals, illiteracy is significantly negatively correlated with height. However, in the case of the Mapuche population, the correlation is entirely reversed. One potential explanation (though certainly not the only explanation) of this owes to selection—with only those who were relatively smaller learning to read, with larger individuals selecting into physical occupations where returns to literacy were smaller. This phenomenon is also backed up when examining occupational categories. Unlike other populations, in the Mapuche population, those working in manual labor are taller, while those working in white-collar areas are shorter: correlations which are entirely reversed in the Spanish/European inmates. It is important to note that average characteristics of these groups in this population are quite different, as documented in Table A2. Given the considerable variation in group composition, as well as the diversity of living conditions and region of provenance at this time, this explanation should be considered as only one of among multiple competing hypotheses.

**Figure 3:**
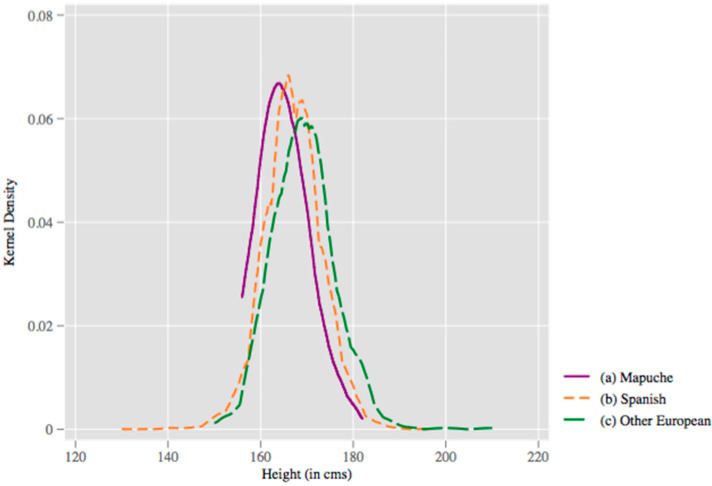
Kernel density of adult male heights for prisoners by ethnicity. Kernel densities of heights are plotted using the full sample of individuals with: (**a**) Mapuche-sounding last names; (**b**) Spanish-sounding last names; and (**c**) other European-sounding last names. Distributions are plotted using a kernel density plot using an Epanechnikov kernel and a bandwidth of 2 cm. Kolmogorov–Smirnov tests reject equality of distributions in each case with *p* < 0.01.

**Figure A2:**
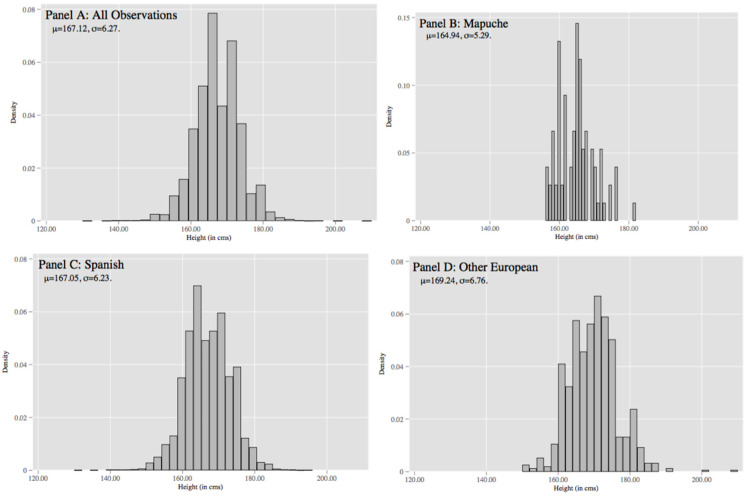
Raw Plots of height by ethnicity. Notes: Raw histograms are displayed for all observations, and each of the groups displayed in Figure 3. Means and standard deviations are displayed in the top left corner of each panel.

Note that all results up to this point refer to correlations with average height in the population, as estimated from standard OLS regression. However, determinants of height may vary considerably over the height distribution, for example being important at low heights and diluted away at higher heights. Prior to examining this in quantile regression, we document that the average height differentials observed in linear regressions are consistent across the distribution of heights, at least in the case of ethnicity indicators. In Figure 3, we document non-parametric kernel density plots of height for the three groups considered in Table 6. Here, we observe that the distributions for Mapuche, Spanish and Other European individuals are entirely horizontally shifted, consistent with height variations at all quantiles of the distribution—short Mapuche individuals are shorter than short Spanish individuals and, similarly, tall Mapuche individuals are shorter than tall Spanish individuals. A similar phenomenon is observed when comparing ethnically Spanish individuals to other European individuals. Formally testing for equality of distributions using a Kolmogorov–Smirnov test suggests these differences are statistically significant (raw distributions are documented in Figure A2).

**Figure 4:**
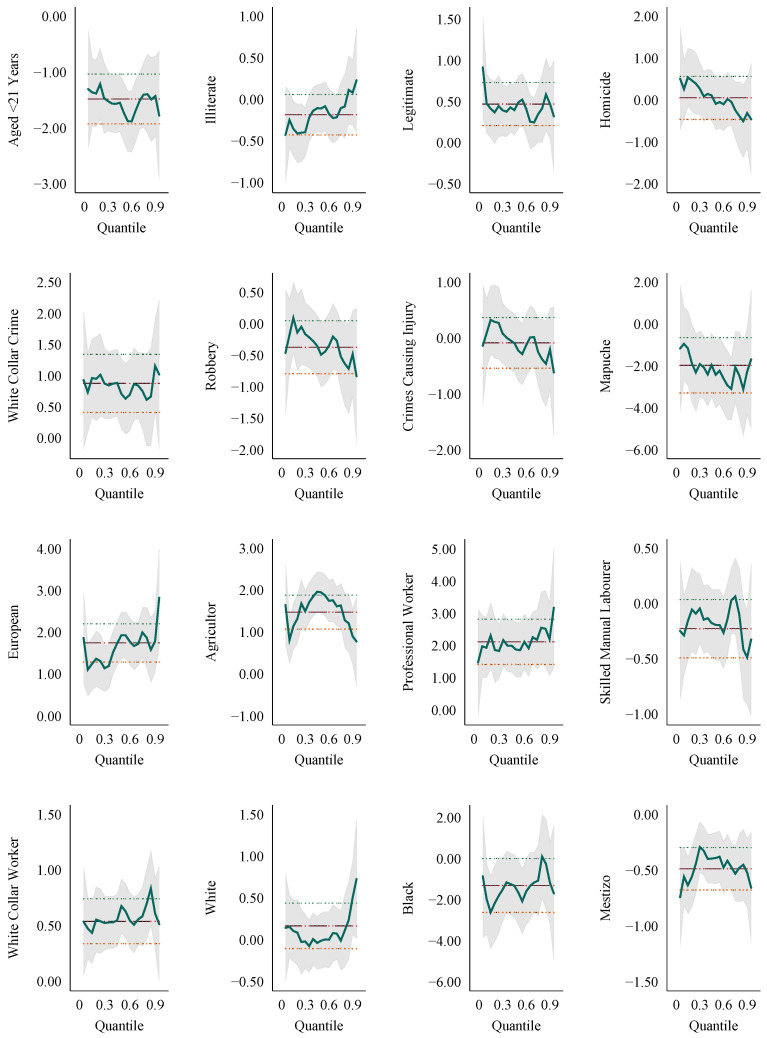
Heterogeneity of correlates over the height distribution. Plots depict coefficient estimates and 95% confidence intervals from quantile regressions. Standard errors are calculated by bootstrap and coefficients are displayed for quantile regressions for each percentile across the height distribution. OLS point estimates and confidence intervals are displayed for reference.

Finally, full distributional analysis is presented in Figure 4. Here, we document the coefficients from quantile regression across the distributions of height. In general, the results suggest considerable stability in these estimates, suggesting that many characteristics are not simply shifting heights at certain parts of the distribution, but rather entirely shifting the distribution. This is the case for young ages, legitimacy, most crime types and occupation classes. Notable exceptions to this pattern are observed when considering literacy, which shifts from being a marginally negative correlate low in the distribution to a marginally positive correlate high in the distribution; Mapuche status, which becomes increasingly negative as increasing in the distribution of heights; and skin colors, where both white and black skin colors are associated with more positive height differentials as heights increase (however, note that, in the case of black individuals, this change ameliorates a negative correlation, while for white individuals it exacerbates a positive correlation).

## 6. Conclusions

Mean height is now widely acknowledged as a good indicator of biological welfare in early infancy that reflects the impact of nutrition, disease environment, work intensity and the socioeconomic condition of people. For countries/periods lacking good data on income distribution and living standards, as is the case for Chile during the 1880s–1930s, adult height is perhaps the best available measure of welfare. Hence, height data provide useful information to policy makers. The WHO and other international agencies have been increasingly using average height as a gauge of nutritional status in developing countries [15,37]. Although the anthropometric history of Chilean adult male height has been fairly well covered before this contribution, previous studies did not contain much information on the role of socioeconomic status on height, which is also the case for many other countries and previous studies based on prison records, thus missing an important part of the overall picture. It is now widely acknowledged that there is a strong connection between socioeconomic conditions and human growth at a household level, and in particular that differences in average height by SESs are a useful indicator of inequality in the biological standard of living.

Consequently, previous studies on Chile accounted for a largely homogenous Chilean adult male population, showing a low level of height inequality per SES. For example, the difference in height per ethnicity in Chile was thought to be smaller than in other Latin American countries for which there is evidence. We aimed to assess the influence of some thus far unexplored or underexplored SES determinants of height for Chile, and in particular to expand on the range of explanatory variables used to assess the main determinants of adult height. We are aware of the fact that our sample may not be entirely representative of the entire Chilean male population, while it ignores the female population. However, our new evidence provides better and newer information on the impact of socioeconomic status on adult male height. Adults with better SES were probably raised as children within households with higher income. There is also evidence that better educated parents usually have healthier and taller children, while it is also believed that the presence of the father, apart from the mother, has a positive impact on people’s height.

Our new data on prison records show that inmates with a university degree were far taller than the rest, which is in line with previous findings for other countries such as the Netherlands, Brazil, Argentina, Mexico, Colombia, Peru and New Zealand. We also found that those legitimately born were taller in adulthood, suggesting that there were differences in well-being between the illegitimate and the legitimately born. This is an important contribution to the literature because this explanatory variable was not used before for developing countries. We also show that those (Chilean born) whose surnames were Northern European were also taller than the rest, and in particular than those with Mapuche background, showing important ethnic inequalities similar to those found, e.g., in New Zealand, Colombia, Mexico, Brazil, Peru and the US. Finally, those able to read and write were also taller than illiterate inmates, as was noted previously for countries such as Switzerland, Ireland, Argentina and Spain. We showed that there was more height inequality in the Chilean population according to socioeconomic status and human capital than previously thought, while we also confirmed the importance of socioeconomic influences during childhood on physical growth. The idea of an exceptionally homogenous Chilean adult male population, based on previous height studies, is not borne out by this new evidence. On the contrary, Chilean height inequality fits better with the experience of other Latin American countries for which there is available height evidence per several socioeconomic variables.

Conditional regression analysis, examining both correlates at the mean and correlates across the height distribution, back up these findings. Legitimate birth status is consistently observed to be associated with greater height at maturity, with strong gradients also observed in ethnically Mapuche, Spanish (Castilian) and Northern European surnames. We observed considerable variation in the correlates of height even within these population groups, suggesting diverse interactions with environmental and socioeconomic factors driven by group status. Overall, conditional regression analysis of this large population suggests considerable and robust socioeconomic variation in heights, both at the mean and across the height distribution.

Future research should try to include female convicts, although we have been unable to locate primary sources to do so. However, we recently managed to find a small collection of female files within military records, which will provide the basis for the first study on sexual stature dimorphism for Chile. Equally important, since we covered only cohorts born during the nitrate era, it would be interesting to see whether the height inequalities we found per socioeconomic status remain the same in previous or later periods. This is important because, for other countries, such as New Zealand, these differentials have been reduced over time.

## Figures and Tables

**Table 1:** Raw average height (cm) per occupation before entering jail (for ages 17–55).

Occupation	Observations	Raw Average Height	Min	Max	SD
Professional (university degree)	325	169.7	144	190	6.4
Farmer (*agricultor*)	1045	168.2	130	190	6.3
White-collar worker	6725	167.6	136	210	6.3
Unskilled manual worker	9349	166.7	140	196	6.2
Skilled manual worker	2839	166.5	140	186	6.1
Unavailable	42		157	183	6.5
Total	20,325	167.1	130	210	6.3

**Table 2:** Raw average height (cm) per literacy level and classification of son at birth (for those aged between 17 and 55 years).

**Literacy**	**Observations**	**Raw Average Height**
Read and write	16,840	167.2
Illiterate	3246	166.8
Just read (does not write)	112	166.2
Unavailable	127	
TOTAL	20,325	167.1
**Sort of son at birth**	**Observations**	**Raw Average Height**
Legitimate	17,786	167.2
Natural	2116	166.6
Illegitimate	224	166.3
Unavailable	199	
Grand Total	20,325	167.1

**Table 3:** Raw average height (cm) per surname’s ethnicity and per skin color (for those ages 17–55), 20,325 observations.

**Surname Ethnicity**	**Obs**	**Raw Average Height**
German	86	172.2
British	176	170.0
Basque	175	168.7
French	124	168.3
Italian	177	167.7
Castilian	19,400	167.1
Mapuche	87	164.9
Others	54	
Unavailable	46	
Grand Total	20,325	167.1
**Skin color**	**Raw Average Height**	**Obs**
*Trigueño*	167.6	63
White	167.5	2601
Dark (*moreno*)	166.7	7923
Black	165.7	89
Unavailable	167.4	9649
Grand Total	167.1	20,325

**Table 4:** Category of crime committed by Santiago Public Jail inmates, of those entering jail between 1936 and 1952 (ages 17–55), 20,325 observations.

Crime Committed	Obs	Shares	Raw Average
White-collar crime	2888	14.2%	168.4
Drunkenness	307	1.5%	168.4
Fire incident	41	0.2%	167.6
Homicide	1491	7.3%	167.3
Causing injuries	3508	17.3%	167.1
Sexual assault	381	1.9%	166.8
Theft	11,510	56.7%	166.8
Others	166	0.8%	167.1
Unavailable	33		166.7
Grand Total	20,325		167.1

**Table 5:** Conditional regressions of height on observed characteristics of prisoners.

Height in cm	(1)	(2)	(3)	(4)	(5)
Aged < 21 Years	−1.723 ***	−1.668 ***	−1.526 ***	−1.480 ***	
	(0.225)	(0.225)	(0.225)	(0.225)	
Legitimate	0.563 ***	0.506 ***	0.461 ***	0.462 ***	0.456 ***
	(0.132)	(0.132)	(0.132)	(0.132)	(0.132)
Illiterate	−0.247 **	−0.256 **	−0.19	−0.204 *	−0.18
	(0.120)	(0.123)	(0.123)	(0.123)	(0.123)
White	0.059	0.11	0.15	0.164	0.197
	(0.138)	(0.137)	(0.137)	(0.137)	(0.137)
Black	−1.561 **	−1.323 **	−1.195 *	−1.177 *	−1.084
	(0.664)	(0.662)	(0.661)	(0.660)	(0.660)
Mestizo	−0.636 ***	−0.559 ***	−0.482 ***	−0.469 ***	−0.461 ***
	(0.095)	(0.095)	(0.096)	(0.096)	(0.096)
Mapuche	−2.044***	−2.097***	−2.073***	−1.733***	−1.721**
	(0.670)	(0.667)	(0.666)	(0.672)	(0.671)
European	2.030 ***	1.891 ***	1.799 ***	1.820 ***	1.811 ***
	(0.232)	(0.231)	(0.231)	(0.231)	(0.231)
*Agricultor*		1.563 ***	1.516 ***	1.516 ***	1.493 ***
		(0.205)	(0.205)	(0.205)	(0.205)
Professional Worker		2.574 ***	2.148 ***	2.161 ***	2.160 ***
		(0.352)	(0.355)	(0.354)	(0.354)
Skilled Manual Laborer		−0.272 **	−0.257 *	−0.271 **	−0.294 **
		(0.133)	(0.133)	(0.133)	(0.133)
White Collar Worker		0.676 ***	0.541 ***	0.558 ***	0.565 ***
		(0.101)	(0.102)	(0.102)	(0.102)
Homicide			0.043	0.039	0.033
			(0.259)	(0.259)	(0.259)
White Collar Crime			0.842 ***	0.825 ***	0.771 ***
			(0.235)	(0.235)	(0.235)
Robbery			−0.386 *	−0.388 *	−0.341
			(0.212)	(0.212)	(0.212)
Crimes Causing Injury			−0.145	−0.166	−0.18
			(0.229)	(0.229)	(0.229)
Constant	166.920 ***	166.630 ***	166.793 ***	167.187 ***	162.379 ***
	(0.135)	(0.143)	(0.248)	(0.359)	(0.643)
Observations	20325	20325	20325	20325	20325
R-Squared	0.012	0.019	0.023	0.026	0.03
Demographics	Y	Y	Y	Y	Y
Occupational Characteristics		Y	Y	Y	Y
Crime Characteristics			Y	Y	Y
Region Fixed Effects				Y	Y
Age Fixed Effects				Y	Y

Notes: Each specification regresses height in centimeters on observable characteristics of the full sample of prisoners. Controls are progressively introduced as indicated in the table footer. The baseline category for occupational groups is “unskilled manual laborer”, the baseline category for crime groups is other crimes (including sexual abuse, drunkenness and causing fires), the baseline for skin color is the group with no color recorded and the baseline for ethnographic background is Arabian. A full set of dummies for age groups are included in Column 5, which also includes dummies for those aged < 21 years. Thus, the omitted baseline group refers to a person of Arabic background, who is literate and illegitimate, aged over 21 years of age and with work type listed as unskilled manual labor. Standard errors are reported in parentheses. * *p* < 0.10, ** *p* < 0.05, *** *p* < 0.01.

**Table 6:** Conditional regressions of height on observed characteristics of prisoners by name origin.

	Mapuche			Spanish			Other European		
	(1)	(2)	(3)	(4)	(5)	(6)	(7)	(8)	(9)
Aged < 21 Years	2.617	1.61	2.249	−1.616 ***	−1.491 ***	−1.451 ***	−1.502	−1.191	−1.249
	(3.549)	(3.843)	(3.794)	(0.227)	(0.227)	(0.227)	(1.437)	(1.424)	(1.433)
Legitimate	−0.454	−0.452	−0.996	0.412 ***	0.371 ***	0.375 ***	4.002 ***	3.568 ***	3.543 ***
	(1.737)	(1.795)	(1.782)	(0.134)	(0.134)	(0.134)	(0.913)	(0.909)	(0.912)
Illiterate	3.169 **	2.964 **	2.478 *	−0.299 **	−0.229 *	−0.246 **	−2.207 **	−1.976 *	−1.897 *
	(1.345)	(1.414)	(1.469)	(0.124)	(0.124)	(0.124)	(1.029)	(1.017)	(1.020)
*Agricultor*	0.765	0.719	−0.822	1.591 ***	1.534 ***	1.533 ***	0.983	1.438	1.526
	(2.129)	(2.216)	(2.268)	(0.208)	(0.209)	(0.209)	(1.247)	(1.239)	(1.241)
Professional Worker	−5.798	−4.608	−6.494	2.913 ***	2.477 ***	2.494 ***	0.968	0.501	0.502
	(4.938)	(5.378)	(5.433)	(0.368)	(0.370)	(0.370)	(1.298)	(1.300)	(1.300)
Skilled Manual Laborer	4.643 **	4.553 *	3.087	−0.256 *	−0.242 *	−0.256 *	−0.841	−0.509	−0.419
	(2.326)	(2.377)	(2.413)	(0.135)	(0.135)	(0.135)	(0.854)	(0.846)	(0.848)
White Collar Worker	−2.505 **	−2.236 *	−3.266 **	0.687 ***	0.545 ***	0.563 ***	1.377 **	1.257 **	1.267 **
	(1.176)	(1.250)	(1.318)	(0.103)	(0.104)	(0.104)	(0.547)	(0.550)	(0.551)
Homicide		−3.282	−4.214		−0.084	−0.091		3.455 **	3.368 **
		(3.137)	(3.113)		(0.265)	(0.265)		(1.440)	(1.443)
White Collar Crime		−2.867	−4.017		0.656 ***	0.641 ***		5.365 ***	5.408 ***
		(3.245)	(3.308)		(0.241)	(0.240)		(1.206)	(1.208)
Robbery		−1.596	−2.349		−0.553 **	−0.553 **		3.401 ***	3.366 ***
		(2.807)	(2.779)		(0.217)	(0.217)		(1.161)	(1.164)
Crimes Causing Injury		−1.134	−1.448		−0.286	−0.308		3.676 ***	3.675 ***
		(2.848)	(2.823)		(0.234)	(0.234)		(1.311)	(1.312)
Constant	165.253 ***	166.926 ***	167.894 ***	166.42 ***	166.846 ***	166.804 ***	165.099 ***	161.695 ***	161.781 ***
	(1.806)	(3.363)	(4.814)	(0.136)	(0.247	(0.707)	(0.913)	(1.426)	(6.706)
Observations	87	87	87	19400	19400	19400	755	755	755
R-Squared	0.22	0.239	0.303	0.012	0.016	0.019	0.053	0.082	0.088
Region Fixed Effects			Y			Y			Y

Notes: Refer to notes to Table 5. This table estimates specifications by name origin (Mapuche, Spanish and Other European) corresponding to Columns 2–4 of Table 5, however removing ethnicity and name origin indicators (given that they are perfectly collinear with the name origin groupings). All other details (including baseline omitted categories) follow Table 5. Standard errors are reported in parentheses. * *p* < 0.10, ** *p* < 0.05, *** *p* < 0.01.

## Appendix A

**Table A1:** Conditional regressions of height on observed characteristics controlling for decade of birth.

Height in cm	(1)	(2)	(3)	(4)	(5)
Born in 1890	0.488	0.49	0.529	0.621	0.553
	(0.412)	(0.411)	(0.410)	(0.410)	(0.468)
Born in 1900	0.457	0.458	0.542	0.651 *	0.668
	(0.394)	(0.393)	(0.393)	(0.393)	(0.510)
Born in 1910	0.323	0.329	0.468	0.615	0.962 *
	(0.389)	(0.388)	(0.387)	(0.388)	(0.533)
Born in 1920	0.018	0.014	0.207	0.389	1.155 **
	(0.394)	(0.393)	(0.393)	(0.394)	(0.551)
Born in 1930	−0.136	−0.139	0.09	0.309	1.351 **
	(0.527)	(0.525)	(0.525)	(0.525)	(0.661)
Aged < 21 Years	−1.615 ***	−1.560 ***	−1.460 ***	−1.437 ***	
	(0.229)	(0.228)	(0.228)	(0.228)	
Legitimate	0.541 ***	0.484 ***	0.447 ***	0.453 ***	0.463 ***
	(0.133)	(0.132)	(0.132)	(0.132)	(0.132)
Illiterate	−0.259 **	−0.269 **	−0.196	−0.205 *	−0.16
	(0.120)	(0.123)	(0.123)	(0.123)	(0.123)
White	0.142	0.195	0.209	0.207	0.115
	(0.140)	(0.139)	(0.139)	(0.139)	(0.141)
Black	−1.491 **	−1.253 *	−1.152 *	−1.148 *	−1.128 *
	(0.664)	(0.662)	(0.661)	(0.660)	(0.660)
Mestizo	−0.572 ***	−0.494 ***	−0.438 ***	−0.437 ***	−0.529 ***
	(0.097)	(0.097)	(0.097)	(0.097)	(0.099)
Mapuche	−1.989 ***	−2.043 ***	−2.032 ***	−1.702 **	−1.749 ***
	(0.670)	(0.667)	(0.666)	(0.672)	(0.672)
European	2.027 ***	1.887 ***	1.799 ***	1.821 ***	1.807 ***
	(0.232)	(0.231)	(0.231)	(0.231)	(0.231)
Farmer *(Agricultor)*		1.558 ***	1.516 ***	1.515 ***	1.490 ***
		(0.205)	(0.205)	(0.205)	(0.205)
Professional Worker		2.565 ***	2.159 ***	2.174 ***	2.148 ***
		(0.352)	(0.355)	(0.354)	(0.355)
Skilled Manual Laborer		−0.288 **	−0.265 **	−0.274 **	−0.287 **
		(0.133)	(0.133)	(0.133)	(0.133)
White Collar Worker		0.675 ***	0.545 ***	0.560 ***	0.568 ***
		(0.101)	(0.102)	(0.102)	(0.102)
Homicide			0.025	0.024	0.053
			(0.259)	(0.259)	(0.259)
White Collar Crime			0.818 ***	0.808 ***	0.772 ***
			(0.235)	(0.235)	(0.235)
Robbery			−0.375 *	−0.382 *	−0.335
			(0.212)	(0.212)	(0.212)
Crimes Causing Injury			−0.158	−0.175	−0.163
			(0.229)	(0.229)	(0.229)
Constant	166.627 ***	166.338 ***	166.371 ***	166.608 ***	161.203 ***
	(0.404)	(0.407)	(0.453)	(0.518)	(0.850)
Observations	20325	20325	20325	20325	20325
R-Squared	0.012	0.02	0.024	0.026	0.03

Notes: Refer to notes to Table 5. Identical regressions are displayed, with identical baseline omitted categories, however now conditioning on decade of birth fixed effects. Standard errors are reported in parentheses. * *p* < 0.10, ** *p* < 0.05, *** *p* < 0.01.

**Table A2:** Descriptive statistics of illiteracy, occupational classes and legitimacy by ethnicity.

	All		Mapuche		Spanish		European		Other	
	Mean	Std. Dev.	Mean	Std. Dev.	Mean	Std. Dev.	Mean	Std. Dev.	Mean	Std. Dev.
Illiterate	0.159	0.366	0.195	0.396	0.16	0.369	0.063	0.243	0.120	0.325
Occupation Groups										
Farmer (*Agricultor*)	0.051	0.220	0.068	0.252	0.05	0.222	0.043	0.203	0.063	0.243
Professional Worker	0.016	0.125	0.011	0.104	0.02	0.122	0.038	0.191	0.000	0.000
Skilled Manual Labourer	0.140	0.347	0.057	0.232	0.14	0.348	0.103	0.304	0.133	0.340
White Collar Worker	0.331	0.471	0.333	0.471	0.33	0.468	0.462	0.499	0.494	0.500
Unskilled Labourer	0.460	0.498	0.529	0.499	0.47	0.499	0.352	0.478	0.301	0.459
Legitimate	0.875	0.331	0.896	0.305	0.873	0.333	0.923	0.267	0.807	0.395
